# “My safe haven turned into a terror zone”: A qualitative study of family members’ experiences of violence by brain tumor patients

**DOI:** 10.1371/journal.pone.0340959

**Published:** 2026-01-27

**Authors:** Amina Guenna Holmgren, Annika Malmström, Eskil Degsell, Johanna Simmons, Lisa Kastbom

**Affiliations:** 1 Department of Neurobiology, Care Sciences and Society, Karolinska Institutet, Stockholm, Sweden; 2 Clinical Department of Geriatrics and Palliative Medicine in Linköping, Region Östergötland, Linköping, Sweden; 3 Division of Cell and Neurobiology, Department of Biomedical and Clinical Sciences, Linköping University, Linköping, Sweden; 4 Stockholm, and Department of Micro, Tumor and Cell Biology (MTC), Karolinska Institutet, Swedish Brain Tumor Association and NOCRiiC, Neuro Oncology Clinical Research, Innovation, Implementation and Collaboration, Karolinska University Hospital, Stockholm, Sweden; 5 Department of Health, Medicine and Caring Sciences, Linköping University, Linköping, Sweden; 6 Primary Health Care Center Ekholmen, Region Östergötland, Linköping, Sweden; Universidad Católica Sedes Sapientiae: Universidad Catolica Sedes Sapientiae, PERU

## Abstract

**Background:**

Knowledge is lacking regarding how the experience of being exposed to violence is affected when the perpetrator suffers from behavioral and personality changes (BPC) due to a brain tumor. This study is part of the Swedish national research project BRAVE - ***B****rain Tumor* ***R****elated* ***A****ggression and* ***V****iolence* ***E****xposure*. The aim was to explore experiences of family members exposed to violence by a person suffering from BPC associated with a brain tumor.

**Methods:**

Individual interviews were conducted with 25 family members who have been exposed to violence by patients with primary brain tumor. The interviews were analyzed using qualitative content analysis.

**Results:**

The participants reported various forms of violence and expressed intense suffering, loneliness and social isolation. The homes sometimes shifted from being a safe place to being a place marked by fear and unpredictability. In adapting to violence, what initially seemed unreasonable, gradually became “the new normal”. Different strategies to minimize risks and damage were described. Self-blame and shame were often associated with an inability to love the patient “in sickness and in health”, despite the violent actions by the patient. When the death of the perpetrator was viewed as the only means of escape, participants also expressed feelings of guilt and shame.

**Conclusions:**

Our study highlights extensive suffering, vulnerability, loneliness and isolation among family members exposed to violence by brain tumor patients. An intervention that provides appropriate support for brain tumor patients, family members and staff who encounter them is urgently needed.

## Introduction

Gliomas are incurable and treatment focuses on prolonging life, managing symptoms and preserving or improving quality of life (QoL). When the tumor progresses, symptoms often increase. Both the disease itself and treatment can lead to neurological, cognitive and psychiatric symptoms [1–3]. Behavioral and personality changes (BPC) caused by gliomas can have major impact on the daily life of both patients and their loved ones [1–3]. Zwinkels et al. conceptualize BPC in adults with glioma as a change of personality and behavior that may result from the tumor itself and/or its treatment. This phenomenon can fluctuate in severity, frequency and magnitude throughout the disease trajectory. BPC encompasses alterations in three domains: (1) *Emotions, needs and impulses*, including loss of emotional control, diminished motivation or initiative, and indifference; (2) *Personality traits*, such as increased selfishness, obsessive tendencies, or rigidity; and (3) *Judgment and decision-making abilities*, characterized by impaired evaluative capacity (3). Though studies exploring this phenomenon are scarce, they indicate that BPC can be both difficult to detect and treat [1]. It has been demonstrated that frontal and temporal tumor location is associated with an increased likelihood of psychiatric symptoms, including BPC [4]. An alarming effect of BPC is the perpetration of violent acts.

In this study, we investigate the experiences of being exposed to violence as a family member of a person with a primary brain tumor, mainly glioma. We include the experiences of both intimate partners and adult children, some of whom were children when the violence occurred. According to the WHO, intimate partner violence (IPV) is defined as ”behavior within an intimate relationship that causes physical, sexual or psychological harm, including acts of physical aggression, sexual coercion, psychological abuse and controlling behaviors. This definition covers violence by both current and former spouses and partners.” [5] Child maltreatment is the abuse and neglect that occurs in children under 18 years of age in the context of a relationship of responsibility, trust or power [6]. Both IPV and child maltreatment are considered serious public health problems resulting in significant social and economic suffering [7,8], as well as long-standing negative health consequences. [8–15].

In recent years, the Swedish Brain Tumor Association and Swedish healthcare providers have noted that BPC related to brain tumors has sometimes led to threats and various types of violence directed toward family members. In the literature, only rare case reports describe the association between brain tumors and violence in close relationships [16–18]. In contrast, the correlation between other types of brain disorders and IPV has been studied to a somewhat larger extent. For example, a correlation has been reported between traumatic brain injury and IPV [19]. For individuals with neurodegenerative diseases, specifically dementia, both exposure to violence and perpetration of violence are common [20].

Knowledge is lacking regarding how the experience of being exposed to violence is affected when the perpetrator suffers from BPC due to primary brain tumor. There is also a lack of guidelines on how support should be provided to this group of victims, as their situation often falls outside of what is typically handled within the healthcare system, social services and legal system. This study is part of the Swedish national research project BRAVE - ***B****rain Tumor* ***R****elated* ***A****ggression and* ***V****iolence* ***E****xposure*. The aim was to explore experiences of family members exposed to violence by a person suffering from BPC associated with a brain tumor, primarily glioma.

## Methods

### Design

A qualitative descriptive design was used to explore the experiences of family members exposed to violence by a person suffering from BPC caused by a brain tumor [21]. To ensure rigorous reporting, the study adhered to the Consolidated Criteria for Reporting Qualitative Research (COREQ) checklist [22].

### Setting and participants

Participants were recruited through purposive sampling by national association announcements via the Swedish Brain Tumor Association and clinical referrals through multiple clinics specializing in brain tumor patient care. The inclusion criteria were: being a family member who had experienced violence perpetrated by a person with glioma, at least 18 years of age at the time of the interview, ability to speak Swedish and consent for the interview to be recorded. Exclusion criteria were: cognitive impairment and/or severe psychiatric illness that would hinder participation in the interview.

The study was performed in line with “World Medical Association Declaration of Helsinki: Research involving human subjects”. Approval was granted by the Swedish Ethical Review Authority (Dnr.: 2023-05969-01). Written informed consent was obtained from each participant before conducting the interview. Four participants provided written consent based on the understanding that their immediate relative suffered from glioma. However, after their interviews were conducted, it was discovered that the patient suffered from meningioma. Despite this, their interviews were included, as their experiences were deemed similar to those of the other participants. The recruitment period was between 26/02/2024 and 08/01/2025.

### Data collection

A total of 25 participants, who were family members of 21 unique brain tumor patients, were interviewed. For descriptive data of the participants and interview details, please see Table 1. An interview guide [23] (see Appendix) was constructed by the research group. The interviews were semi-structured with open-ended questions. After an opening presentation of the study, the participants were asked to share their experiences of the violence they had encountered. The researcher who conducted the interviews is a clinically active general practitioner, routinely working with patients suffering from severe illnesses and their family members. She is accustomed to managing conversations on sensitive and challenging topics and was attentive and responsive to participants’ emotional reactions throughout the interviews. When participants exhibited signs of distress, appropriate individual support was offered, including options to pause or end the interview, as well as referral to relevant services. If participants disclosed a current risk of violence, they were offered referral to healthcare services, national helplines for victims of violence and crime, or a private telephone follow-up with the interviewer. Additionally, an information sheet listing important telephone numbers for various support services, specialized in assisting individuals exposed to violence, was provided as an appendix to the written participant information and consent form to all the participants prior to each interview. The interviews were digitally recorded and were transcribed verbatim by an external secretarial service. None of the researchers in the research group were involved in the care or treatment of the relatives who suffered from glioma or meningioma.

**Table 1:** Descriptive data of the 25 participants and interview details.

	n	%
**Age at the time of interview**		
**-** mean (years; range)	50 (21–75)	
- median (years)	56	
**Gender**		
**-** female	20	80
- male	5	20
**Relation to the brain tumor patient**		
**-** partner	21	84
- child	4	16
**Status of the brain tumor patient**		
**-** alive	7	33
- deceased	14	67
Duration of illness*		
**-** < 1 year	9	36
− 1–5 years	11	44
− 6–10 years	3	12
- > 10 years	2	8
**Living situation at the time of the interview**		
**-** living with another adult	4	16
**-** living with another adult and child/children	3	12
- living with child/children	6	24
- living alone	12	48
**Participant recruited from**		
**-** healthcare	14	56
- the Swedish Brain Tumor Association	11	44
**Interview details**		
**-** time of interviews	Feb 2024-Jan 2025	
- duration of interview	58–148 minutes	
- interview setting		
• telephone	9	
• online	9	
• physical meeting	7	

*) When patient deceased: Time from diagnosis until death.

When patient alive: Time since diagnosis at the time of the interview.

### Analysis

**Fig 1:**
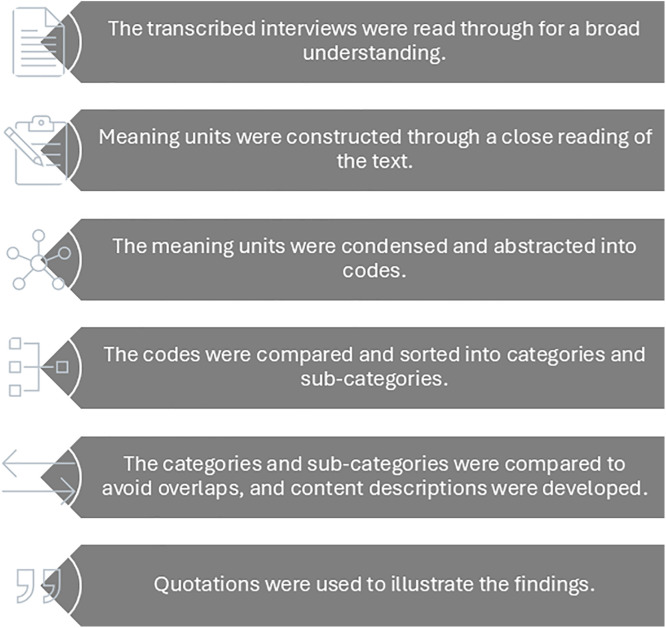
The six steps used in the qualitative content analysis according to Graneheim & Lundman.

The data was analyzed using qualitative content analysis without predetermined categories (see Fig 1) [24]. Initially, two researchers with experiences in qualitative research (AGH: PhD, nurse, and LK: PhD, physician) independently read all 25 transcribed interviews to gain an individual understanding and a general overview of the participants’ experiences. After the initial reading and the first analysis, AGH and LK systematically analyzed the material. Subsequently, the entire research group discussed the categories and subcategories until a consensus was reached. As part of the reflexivity process, the categories were validated by supplementing and contesting each other’s interpretations and preunderstandings [23]. Quotes were selected to illuminate the findings and make it easier for readers to understand the main content, in this manuscript focusing on experiences.

## Results

**Fig 2:**
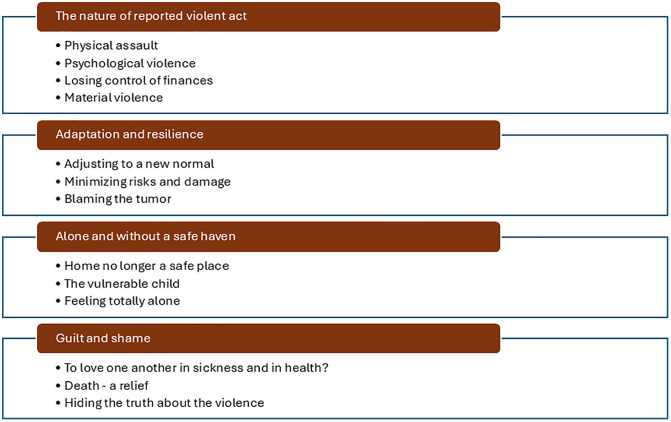
Overview of the four categories with thirteen sub-categories describing experiences of family members exposed to violence by a person suffering from BPC due to a brain tumor in a Swedish context.

The data analysis resulted in the identification of four categories and thirteen sub-categories describing the experiences of family members exposed to violence by a person suffering from BPC related to a brain tumor (see Fig 2). Experiences from family members of both deceased and living patients were analyzed collectively, with data from family members representing both groups evident across all the categories. A qualitative comparison revealed no significant differences. To ensure confidentiality, participant characteristics shown in Table 1 are only presented at group level.

Table 2 summerizes the categories and sub-categories with example quotations.

**Table 2:** Summary of the four categories and thirteen subcategories, including quotations that exemplify the findings.

Quotation	Sub-category	Category
*“Our then two and a half year old had gotten out of the car and I saw that he was there pulling on our son’s arm, so I simply asked, “What are you doing?” “What about it?” “Well, you’re pulling too hard,” “No, no, I just want to hear what it sounds like when it breaks.”*	Physical assault	The nature of reported violent act
*“Then he developed a personality where he wanted to fight the disease. And I became the disease. I personified what he was suffering from. Which made him fight … try to fight me.”*	Psychological violence	
*“Because I will be in a situation where I alone will have to take over all the mortgages and while she is sitting there in her chair happily promising to actually give money to our son…// This is what I’m thinking; what happens if she suddenly decides to give all assets to our son as a gift…”?*	Losing control of finances	
*“Then he pushed that knife right through the cutting board, so that there was a hole in our kitchen counter too. So, he really got it in there, although luckily he didn’t end up hurting anyone.”*	Material violence	
*“Then you thought: this is completely crazy. But after a while it becomes normal. You get used to “now he’s like this”, and then you go to the next step.”*	Adjusting to a new normal	Adaptation and resilience
*“I learned to just keep quiet and bite my tongue because it was easier that way. If I just be quiet and bite my tongue and put on a nice face, then it will be fine.”*	Minimizing risks and damage	
*“Then it’s like, if you stand behind a person who is terminally ill, you allow that person to do a lot more things that you might not have done otherwise. Because you think like,’God, he’s dying, so I can’t be mad at him’.”*	Blaming the tumor	
*“What was supposed to be my home, my fortress, my safe place, was instead a terror zone.”*	Home no longer a safe place	Alone and without a safe haven
*“Then when Dad got so mean, I took Mom’s side, and I intervened several times when they were arguing, when he was hitting her too. So, I think that’s why he felt that I... or it divided us a little bit and that we argued a little more intensely.”*	The vulnerable child	
*“And I talked to some of our friends and they didn’t believe me. Well, he was the nicest guy in the world and I was clearly being too sensitive.”*	Feeling totally alone	
*“But I still needed to protect myself and my son from it and at the same time care for him as sick as he was, the one I had promised to love now and always.”*	To love one another in sickness and in health?	Guilt and shame
*“Can mom die soon? We don’t want mom here on Christmas Eve. I want to open the packages without mom. I’d like to sit up in the room and open the packages. I don’t want mom here. We think it’s hard. Can’t mom go to the hospital.”*	Death – a relief	
*“So, I got up earlier and I made sure that if I had slept in shorts or something like that and I had ugly marks on me, then I made sure that I had long pants and long sleeves on when they came over. Then I wouldn’t be walking around in shorts and a t-shirt.”*	Hiding the truth about the violence	

### The nature of reported violent act

#### Physical assault.

Participants described experiencing various forms of physical violence, including kicking, pushing, strangulation, hitting and biting. In some cases, the violence was direct, while in others, it was more subtle. In some families, both the healthy partner and children became targets of the violence. Participants described incidents of severe physical violence, sometimes requiring police intervention and medical assessment. Exposure to violence resulted in fear of losing their lives or suffering from serious injuries.

*“Our then two and a half year old had gotten out of the car and I saw that he was there pulling on our son’s arm, so I simply asked, “What are you doing?” “What about it?” “Well, you’re pulling too hard,” “No, no, I just want to hear what it sounds like when it breaks.”*

(Participant 3. Partner)

The unpredictability of the violence left participants in a constant state of vigilance. While some physical violence was deliberate, others described situations where the ill partner seemed unaware of their actions, for example gripping the healthy partner’s arms too tightly, leaving bruises, or using them for support when unsteady, resulting in injury. Despite the lack of intent in some cases, the consequences were still painful and frightening for those affected. Family pets were also occasional victims of violence, sometimes being kicked or hit. In addition to harming others, the ill partner sometimes engaged in self-destructive behavior, such as hitting themselves or banging their heads against walls. Efforts to intervene and prevent self-harm resulted in the healthy partner being physically assaulted.

#### Psychological violence.

Psychological violence affected the families’ daily lives and emotional well-being. Participants described how their needs and emotions were gradually disregarded, leaving them feeling invisible and unimportant. They were often accused of being the cause of the illness, with any difficulties experienced being attributed to the healthy partner or the children.

*“Then he developed a personality where he wanted to fight the disease. And I became the disease. I personified what he was suffering from. Which made him fight … try to fight me.”*

(Participant 1. Partner)

The brain tumor patient often aimed to control the family’s social interactions, limiting their ability to seek support. However, irrational behaviors were also common, and family members reported that the patient would become angry for no reason, displaying irrational jealousy and insisting that no one cared about them. Accusations of infidelity, financial theft and betrayal were common. Verbal abuse played a significant role in the psychological violence experienced by family members. One child recalled:

*“Then he could get really angry and say that... well, that I hurt everyone around me. Or that... umm... well, there was a lot of talk about me being stupid or that I hurt others or that I’m not smart enough or... and stuff like that.”*

(Participant 15. Child)

The fear that violence might escalate from psychological aggression to physical harm was a concern for participants. A recurring fear was that the ill partner might suddenly become aggressive when handling knives or other sharp objects. The unpredictability of their behavior meant that even routine activities, such as cooking, could evoke unease. This fear was magnified when children were present, as participants worried not only for their own safety but also for the well-being of their children. They worried about reckless behavior, such as driving despite medical restrictions, which put their children at risk. When children were left alone with the ill parent, the healthy parent frequently called to check on them, fearing for their emotional and physical safety. Threats directed toward family members sometimes escalated to life-threatening levels. One participant described how the partner threatened to shoot them, while another recalled an incident where their child was threatened with a knife. Such experiences left families in a state of constant distress, as they never knew which version of the ill individual they would encounter.

*“I was afraid he would hit me… That it wouldn’t be enough to just bang down... or hit the table when I was sitting across from him, but that it would end up being me instead.”*

(Participant 18. Partner)

#### Losing control of finances.

The illness impaired the affected individuals’ ability to manage and control money, leading to financial instability that placed additional strain on the families. Participants described how their partners had difficulties remembering purchases and assessing the financial consequences, resulting in excessive spending on unnecessary items. Beyond compulsive shopping, participants revealed that their partners made impulsive financial decisions, such as giving away large amounts of shared savings as early inheritances.

*“Because I will be in a situation where I alone will have to take over all the mortgages and while she is sitting there in her chair happily promising to actually give money to our son…// This is what I’m thinking; what happens if she suddenly decides to give all assets to our son as a gift…”?*

(Participant 17. Partner)

Gambling was another major issue that escalated following diagnosis, leading to severe financial consequences, and forcing one family to sell their shared home to cover gambling debts.

While some ill partners exhibited reckless spending behavior, others became more controlling over financial matters. Participants described being denied sufficient funds for necessities, such as food. Financial disputes could extend beyond the duration of the partnership. In divorce proceedings, one participant encountered difficulties when the former partner refused to honor agreements regarding the division of assets.

To cope with financial instability, participants were forced to remain cautious, meticulously checking transactions and ensuring that bills were paid on time. Others found it necessary to separate their finances from the ill partner to regain some control. Some participants expressed anxiety about financial repercussions, including the risk of accumulating debt and facing legal consequences, such as enforcement orders from debt collection agencies.

#### Material violence.

Participants also reported encountering material violence, which was a form of aggression characterized by the destruction of objects. Participants described situations where doors were slammed with excessive force, walls were struck during moments of anger and objects were thrown, sometimes directly at them. Some described how their partner, during conflicts, inflicted structural damage to the home, such as breaking down doors. These actions not only resulted in material loss but also contributed to a persistent sense of insecurity. In some instances, weapons were used, further intensifying the perceived threat.

*“Then he pushed that knife right through the cutting board, so that there was a hole in our kitchen counter too. So, he really got it in there, although luckily he didn’t end up hurting anyone.”*

(Participant 6. Partner)

### Adaptation and resilience

#### Adjusting to a new normal.

What initially seemed unreasonable and incomprehensible gradually became “the new normal” as the participants adapted to the circumstances. Over time, behaviors and actions once deemed unacceptable were tolerated. Participants described how their partner or parent underwent drastic BPC due to the brain tumor. Violent behavior, which had not previously been present, emerged alongside the progression of the illness.

*“I feel like I lost my partner several years ago and grieved early on. Because there’s only a shell left. Not a semblance of the person I fell in love with and was with, and had children with.”*

(Participant 23. Partner)

The person they had once loved seemed to have disappeared, replaced by someone whose actions were controlled by the tumor. Several participants experienced physical violence that initially made them fear for their lives. However, as time passed, they became accustomed to it, and their perception of its severity changed. Over time, their initial resistance was replaced by a new understanding, where the once unthinkable became part of their everyday reality.

*“Then you thought: this is completely crazy. But after a while it becomes normal. You get used to “now he’s like this”, and then you go to the next step.”*

(Participant 2. Partner)

#### Minimizing risks and damage.

Participants described adopting various strategies to minimize risks and avoid escalating conflicts with their ill partners. A central approach was choosing to go along with the ill partner’s wishes rather than challenging or contradicting them. They learned that resistance often led to intensified aggression, and as a result, they prioritized maintaining peace at the cost of their own needs and well-being. One key strategy was silence. Several participants described how they deliberately refrained from expressing opinions, asking questions or making requests, knowing that any perceived challenge could trigger anger or retaliation.

*“I learned to just keep quiet and bite my tongue because it was easier that way. If I just be quiet and bite my tongue and put on a nice face, then it will be fine.”*

(Participant 8. Partner)

Participants described a need to constantly monitor their partner’s mood and adjust their behavior to avoid conflicts, for example, by staying quiet, avoiding eye contact or leaving the room to prevent escalation. Many expressed a need to analyze their own behavior and actions, trying to understand what they may have done to trigger violent behavior.

*“And I feel that I myself have felt that I am going crazy, because of everything that has happened and think’Am I the one who is wrong? What am I doing wrong?’”*

(Participant 13. Partner)

#### Blaming the tumor.

A recurring theme was that the perpetrator was ill and therefore not fully aware of his or her actions. This reasoning contributed to a growing sense of acceptance, as participants rationalized the violence as a consequence of the perpetrator’s condition, rather than a deliberate act of harm.

*“Then it’s like, if you stand behind a person who is terminally ill, you allow that person to do a lot more things that you might not have done otherwise. Because you think like,’God, he’s dying, so I can’t be mad at him’.”*

(Participant 21, Partner)

Most participants strongly believed that the affected individual would never have engaged in such aggressive or harmful behavior had it not been for the behavioral and personality changes caused by the tumor. However, some indicated that there were pre-existing tendencies toward such behavior, which the tumor appeared to exacerbate. Additionally, participants believed that if the affected individuals had been fully aware of their actions, they would have experienced significant distress over their own behavior.

Beyond the participants’ tendency to attribute violent behavior to the tumor, other people in their social circles frequently used the tumor as an explanatory model to justify or mitigate the severity of the affected individual’s actions. Participants also expressed that had the individual not been ill, they would not have tolerated the same behavior.

*“I would never have wanted to be with him, and definitely not have had children with someone like that. Never. He has changed so much.”*

(Participant 18. Partner)

### Alone and without a safe haven

#### Home no longer a safe place.

A profound shift in the home environment was described by the participants, where a place of safety and comfort instead turned into a source of fear and unpredictability. Their homes were increasingly perceived as a space of confinement and distress.

*“What was supposed to be my home, my fortress, my safe place, was instead a terror zone.”*

(Participant 3. Partner)

Families reported living in a state of constant vigilance, as the ill individual frequently directed aggression toward those who were closest to them, including partners, children and pets. The ill individual sometimes maintained composure at work, among friends or with healthcare staff. As a result, participants felt a lack of recognition and support from others outside the family. This capacity for selective control over violent behavior was particularly distressing for the participants.

*“So, he worked a lot and worked really hard. And what was a bit difficult at the time was to see him work, try to be as competent as he could be and then come home and express himself in this way, because then you feel that … well, if you can act one way in this place, then you must be able to act this way at home too.”*

(Participant 15. Child)

Conversely, in some cases, participants also described acts of aggression toward strangers, which made them feel even more insecure and uncertain.

#### The vulnerable child.

The demands of caring for an ill partner while raising a child often led to conflicts. The parents who participated in the study identified their primary role as protecting their children. However, they had to simultaneously accommodate the needs of the ill partner, leading to challenging situations. Despite their efforts, children frequently perceived these protective measures as inadequate. The children who participated in the study sometimes expressed feelings of being forced to mature prematurely and assume excessive responsibilities compared to their peers. Children struggled with the psychological toll, experiencing difficulties with concentration, memory and academic performance.

The home was described as an increasingly unsafe place for children, who in some cases attempted to avoid the home environment to escape from the ill parent’s violent behavior, while simultaneously trying to protect the healthy parent. The presence of children influenced the behavior of the ill parent in varying ways. In some cases, it appeared to have a calming effect, whereas in others, it exacerbated anger and aggression. Notably, some children who intervened to protect the healthy parent reported experiencing verbal threats and physical intimidation.

*“Then when Dad got so mean, I took Mom’s side, and I intervened several times when they were arguing, when he was hitting her too. So, I think that’s why he felt that I... or it divided us a little bit and that we argued a little more intensely.”*

(Participant 15. Child)

While some children actively avoided their home, others were left in the care of the ill parent due to the work obligations of the healthy parent. The COVID-19 pandemic further exacerbated these challenges, as school closures necessitated remote learning, thereby increasing the children’s exposure to the home environment with the ill parent.

#### Feeling totally alone.

Feelings of loneliness and social isolation were frequent and excessive. As violence primarily occurred at home and was directed toward family members, participants often viewed it as a private issue that they were responsible for managing themselves. This sense of personal responsibility, combined with the controlling behavior of the ill individual, restricted their ability to seek support. Many explained that the affected person always wanted to be present during interactions with others, which made it difficult to seek help or share their experiences without them knowing. Some reflected on how they had no one to rely on and were compelled to navigate their situation on their own. Several noted the disbelief expressed by those who later learned about their situation, emphasizing how outsiders often failed to recognize the severity of their situation.

*“And I talked to some of our friends and they didn’t believe me. Well, he was the nicest guy in the world and I was clearly being too sensitive.”*

(Participant 18. Partner)

Additionally, the emotional detachment from the ill individual further contributed to feelings of isolation. Many expressed that they could no longer share their life with their partner, or parent as they used to, intensifying feelings of loneliness. This shift in the relationship reinforced the sense of abandonment, as participants described losing both emotional and practical support.

### Guilt and shame

#### To love one another in sickness and in health?.

Participants stated that the illness altered their relationship with the affected individual. Many described an inability to love the ill person in the same way as before, which was accompanied by feelings of guilt and shame. The person they had once cherished no longer seemed present and instead exhibited harmful behaviors toward them. Despite this, participants felt unable to leave, feeling they had an obligation to care for the ill person.

*“But I still needed to protect myself and my son from it and at the same time care for him as sick as he was, the one I had promised to love now and always.”*

(Participant 3. Partner)

While many wished for the ill person to move to a nursing home, they found it difficult to make this decision. They also emphasized that there were limits to the mistreatment they could accept, with physical violence toward themselves or a child often identified as a threshold that, if crossed, would prompt them to leave. However, many families who experienced physical violence still chose to stay, as they struggled to envision how the ill person would manage independently.

Participants described a profound sense of guilt and moral obligation in relation to their ill partner. Despite the psychological and emotional burden, the participants often suppressed their own suffering, believing that the person with the illness was the one who was truly struggling. Many expressed the belief that their own difficulties were insignificant in comparison.

*“Because I probably felt like, ’well, he’s the one who’s sick and he’s the one who’s going to die, then I can’t...’ Then it’s like I have to take it, because there is mostly pity for him. So that probably mostly describes my thinking”*

(Participant 9. Partner)

#### Death – a relief.

Some participants described deeply conflicting emotions regarding the impending death of their ill partner. They acknowledged the immense emotional burden of these thoughts, yet for many, death was seen as the only possible release from their own suffering and the suffering of the ill individual. Some expressed the belief that if their partner had been their former, healthy self, they too would have wished for an end to their suffering. The progression of the illness had transformed both their relationship and the person they once loved.

*“But in the same way, it almost feels like I want my husband to die and it’s terrible because deep down I love him. Because he’s an incredibly nice and funny person. He’s still the love of my life. But I just feel like I can’t take it for much longer.”*

(Participant 11. Partner)

This internal conflict was further intensified by feelings of guilt. Some confessed that they never wanted a divorce but had instead longed for their partner’s death as the only escape from their situation. They struggled with feelings of grief and shame, especially when their partner expressed hope for positive medical outcomes, while they secretly wished for the opposite. The weight of this guilt was described as nearly unbearable. They also questioned the necessity of treating the tumor.

*“And sometimes I could even ask... why do treatment? Why prolong this horrible period that destroys [us].”*

(Participant 6. Partner)

For some, death brought a sense of relief. They recounted how they had been subjected to mistreatment and emotional distress for so long that the passing of their partner lifted an overwhelming burden. The deterioration of their partner’s condition brought a sense of respite, as moments of conflict and aggression became less frequent when the ill individual lacked the energy to argue or lash out. One parent whose partner was ill recalled their children expressing wishes for the ill parent’s death, believing it would finally bring peace to the family:

*“Can mom die soon? We don’t want mom here on Christmas Eve. I want to open the packages without mom. I’d like to sit up in the room and open the packages. I don’t want mom here. We think it’s hard. Can’t mom go to the hospital.”*

(Participant 23. Partner)

#### Hiding the truth about the violence.

Participants described a deep reluctance to disclose the violence they experienced, often prioritizing the protection of the ill individual’s reputation over their own safety and well-being. Many feared that revealing the violence could negatively impact the patient’s medical treatment or how they were perceived by healthcare staff. Others hesitated to share their experiences due to shame – in some cases for staying in the situation and sometimes for being subjected to violence.

The stigma and distress associated with their home situation caused the children to refrain from discussing their experiences with others. When they tried to open up, they were often met with doubt or dismissal. One participant, for instance, shared how she confided in a family friend, but ended up not being believed and feeling accused of being ungrateful.

A recurring theme was the feeling of loyalty toward the individual who was severely ill. Some had explicitly promised never to reveal what was happening, creating an internal conflict between honoring that commitment and seeking help. Participants also described efforts to conceal physical signs of violence.

*“So, I got up earlier and I made sure that if I had slept in shorts or something like that and I had ugly marks on me, then I made sure that I had long pants and long sleeves on when they came over. Then I wouldn’t be walking around in shorts and a t-shirt.”*

(Participant 21. Partner)

The need to protect others from the truth also played a role in the participants’ decision not to disclose the violence. Some refrained from sharing their experiences to shield friends and family. They wanted to preserve the good memories of their loved ones rather than allowing the violence to define their relationships.

## Discussion

Patients with a brain tumor can suffer from BPC where loss of impulse control or violent behavior can be a presenting symptom or develop along the disease trajectory. Those caring for these patients in their profession need to be aware that this is part of the clinical reality although rarely acknowledged. There is a need to have the possibility of the occurrence of violence in mind and actively monitor for it, including asking family members.

This study highlights the presence of various forms of violence directed at family members of brain tumor patients, as well as the extensive suffering, vulnerability and loneliness among this group. According to the findings, the onset or escalation of such violence occurred following the brain tumor diagnosis. BPC may result directly from the brain tumor [1]. Also, particularly in cases of high-grade glioma, steroid treatment, commonly used to manage symptoms caused by peritumoral edema, could contribute as it is known to affect behavior [1,25]. Additionally, antiepileptic drugs can provoke aggression as a side effect [1,25–27]. The psychological impact of confronting a life-threatening illness [1,28] may further contribute to emotional instability, which often manifests as irritability, jealousy or loss of impulse control. While certain clinical situations in brain tumor patients may increase the likelihood of BPC, such as frontal or temporal involvement [4,29,30], mass effect or edema [31], corticosteroid use [1,25], antiepileptic medication [1,25–27], perioperative states [32], or disease progression [1], healthcare professionals should remain vigilant for its potential presence and associated consequences, including aggression, threats and violence, in all interactions with patients and their families. In addition, clinicians should be attentive to warning signs that may indicate violence and proactively inquire about the patient’s condition, its impact on the patient, and its effects on family members, and fulfill the mandatory duty to report to child protective services in cases where child safety is at risk. Despite the fact that BPC is well documented [1–3,33] in brain tumor patients, the impact on family members remains underreported.

Another key finding of our study is the multifaceted experiences of violence among family members of brain tumor patients with BPC. In part, our findings are similar to previous research about women’s experiences of IPV, as well as child maltreatment; however, in other respects, our findings are very different. Controlling behaviors are a core component of IPV [34,35]. In this study, controlling behaviors were displayed by the ill person, but a more prominent finding was that family members described a loss of power and control due to irrational and unpredictable behaviors associated with BPC. However, the consequences for the victims were similar as for other victims of IPV, for example, living in a constant fear of psychological and physical violence and losing control of their financial situation.

Feelings of self-blame and shame were prominent in the participants’ narratives, consistent with findings in previous research showing that victims of both IPV and childhood maltreatment often experience self-blame and shame [33,34,36,37]. In this study, such emotions were further intensified by the presence of the brain tumor, as family members expressed guilt over their inability to offer unconditional support and their failure to love the individual “in sickness and in health.” Additionally, they described being unable to leave the abusive relationship because the perpetrator was ill and in need of care. Similarly, in a study including the lived experience of women subjected to dementia-related violence by previously non-violent partners, participants described the need for endurance and prioritizing caring for the partner over their own safety and wellbeing [38]. Partners also described how they gradually accepted violent behavior from the ill person and remained in the relationship even when previously established lines for acceptable behaviors had been crossed. While a gradual adaptation to, and normalization of violent behaviors is common among survivors of childhood maltreatment and IPV [33,39], these tendencies may have been intensified in the present study, as brain tumors may compel both the patient and family members to adapt to a broader new normal. Understanding this dynamic enables healthcare providers, social care and civil society to respond with sensitivity, while ensuring safety and offering timely interventions that address both the physical and psychological needs of those affected, both patient and family member.

Awareness of the brain tumor influenced how others perceived the patient’s violent behavior. The tumor affected societal reactions, with some people dismissing the family members’ suffering and shaming them for acknowledging their own pain. There is a tendency towards societal “othering” of perpetrators of IPV – describing them as deviant from the majority population, sometimes even dehumanized or depicted as monstrous [40,41]. Such societal perceptions may make it difficult for all involved parties to acknowledge that the ill individual is both a victim of a brain tumor and a perpetrator of violence. The perceived need to protect the perpetrator from the negative consequences of disclosure, preserve the image of the aggressor as a “good person” and protect others (children or other relatives) from knowing that their loved one is a perpetrator of violence has previously been described in relation to IPV and child maltreatment [36,37,42]. A similar picture is presented in one of the few case reports found in the literature, where a woman suffering from a brain tumor subjects her husband to severe violence. Despite his difficult situation, the husband is still greatly concerned about the woman’s well-being [17]. Altogether, this underlines the extreme difficulties of living with violence perpetrated by a partner or parent with a severe disease and emphasizes the urgent need for developing a strong societal support and response system. Raising awareness and acknowledging that violence may occur is essential in supporting both the patient and their family members. When encountering violent behavior, it is important for healthcare staff to carefully assess the underlying causes of BPC and take appropriate action. This may involve adjusting medication, providing psychological support to the patient and ensuring that family members receive adequate support. In some cases, this may include arranging for the patient to receive care in a more suitable setting, such as a nursing home, or moving family members to safe shelters.

### Strengths and limitations

The broad variation of participants (in terms of gender, age, living situation, relationship to the individual with a brain tumor, educational level of the participant, method of recruitment and time since diagnosis) supports the transferability of the findings [23], despite an evident risk of selection bias, such as an overrepresentation of individuals engaged with the Swedish Brain Tumor Association, or those experiencing significant severe violence. Furthermore, as this study was conducted in Sweden, where help-seeking pathways may differ from those in other countries, the transferability of the results may be context-dependent. Sexual violence was not mentioned by participants in this study, which is a bit surprising. The reason for this can only be speculated upon, but it may be that this type of violence is particularly shameful. The aim of the study was to focus on violence perpetrated by glioma patients, but several of the participants were family members of a person with meningioma. Still, we could not identify any distinct differences in their experiences compared to participants with a relative suffering of glioma. The involvement of researchers with experience in qualitative research representing different professions and disciplines (nurses and physicians specializing in neuro-oncology, neuro-surgery, geriatrics, palliative medicine and primary care), as well as experiences from engagement in the Swedish Brain Tumor Association, provided an opportunity to validate the findings [23]. The researcher who conducted the interviews is a clinically active physician, which could have influenced the answers of the participants. However, the interviewer attempted to remain neutral throughout the study process and was not involved in the treatment or care of the participants. Consolidated criteria for reporting qualitative research (COREQ) [22] were used for reporting the results, strengthening the trustworthiness of our study.

### Future research directions

Research aiming to explore family members’ experiences of *support or lack of support* from healthcare and society regarding violence is needed and ongoing. There is also a lack of guidelines on how support should be provided to this group of victims, as their situation often falls outside of what is typically handled by the healthcare system, social services and legal system. There is an urgent need to increase knowledge of violence perpetrated by patients suffering from brain tumors. This should include different perspectives and aim to develop an intervention that provides appropriate support for the patients, family members and staff who encounter them.

### Clinical implications

Although the aim of this study was to explore the experiences, rather than the support needs, of family members affected by violence perpetrated by patients with brain tumors, several general clinical recommendations for healthcare professionals can be proposed:

- *Conduct direct and private inquiries* with family members, separately from the patient, regarding the presence of aggression, threats and violence, as well as overall family safety.
- *Establish collaboration with supportive services* and ensure clear referral pathways to social work and specialized violence support services.
- *Fulfill the mandatory duty to report* to child protective services in cases where child safety is at risk.

Importantly, an ongoing study is being conducted to more precisely identify the specific needs of family members exposed to violence, thereby providing a stronger evidence base for developing clear and targeted clinical recommendations.

## Conclusion

Our study stresses the extensive suffering, vulnerability, loneliness and isolation among family members exposed to various forms of violence where brain tumor patients became the perpetrators.

## Supporting information

S1 Appendix Interview guide. (DOCX)

S1 File PLOS One human subjects research checklist. (DOCX)
