# Supplementary material for: “My safe haven turned into a terror zone”: A qualitative study of family members’ experiences of violence by brain tumor patients
Source: PLoS One. 2026 Jan 27;21(1):e0340959. doi: 10.1371/journal.pone.0340959 (PMC12843534; doi:10.1371/journal.pone.0340959)
Supplement: S1 Appendix — (DOCX) [file pone.0340959.s001.docx]

**Interview guide: “My safe haven turned into a terror zone”: a qualitative study of family members’ experiences of violence by brain tumor patients**

- Age
- Sex
- Social situation/family circumstances (living alone, living together, partner, children)
- Relationship to the brain tumor patient
- Educational level/profession

Opening question: As you know, this study is about family members who have been exposed to different types of violence by an individual suffering from a brain tumor. This can involve a number of different types of events, and we would like to understand more about the situations you encounter as a family member. I would therefore like to ask you to tell me about your experience.

- Can you describe how it started?
- How did it change?
- When did it happen?
- What was your relationship like before your loved one was diagnosed with a brain tumor? Were there tendencies towards similar behaviors before they became ill?
- How has your loved one changed due to the illness (brain tumor)?
- How long has the violence occurred, and is it still occurring?
- How do you think what you have been through has affected you? Others around you?
- What type of help/support have you sought? With whom?
- If you have sought help/support:
  - What made you seek help/support?
  - Where did you seek help/support?
  - What was your experience of the help/support you received?
  - What kind of help would you have liked to receive?
  - Did you seek help/support from healthcare providers?
    - Where? When? What was your experience of the help/support you received? Was there anything that was particularly helpful? Is there anything that you feel needs to be improved/changed?
- If you have not sought help/support:
  - What are the reasons you did not seek help/support?
  - What type of help/support would you have liked to receive?
  - How do you feel today about the fact that you did not seek help/support?
  - What could have made you seek help/support?
- How would you like the healthcare system/society to respond to violence?
  - On whose initiative do you think the issue of exposure to violence among family members of people with brain tumors should be raised? Explain why you feel this way.
  - If a family member raises the issue of exposure to violence, what kind of help do you think should be offered? Explain why you feel this way.
  - What are your thoughts on exposure to violence among children of people with brain tumors?
    - How should exposure to violence in children be detected? By whom?
    - Is there anything that you feel is particularly important when it comes to exposure to violence in children?
    - What do you think is the best way to help/support these children?

Forward-looking and concluding question:

- Is there anything else you would like to add?
- What would you like to add regarding your experience of being a family member who has been subjected to violence by an individual suffering from a brain tumor?
